# Mechanism of Magnetic Permeability Perturbation in Magnetizing-Based Eddy Current Nondestructive Testing

**DOI:** 10.3390/s22072503

**Published:** 2022-03-25

**Authors:** Zhiyang Deng, Zhiheng Yu, Zhongyu Yuan, Xiaochun Song, Yihua Kang

**Affiliations:** 1 Key Laboratory of Modern Manufacture Quality Engineering, Hubei University of Technology, Wuhan 430068, China; 101910036@hbut.edu.cn (Z.Y.); 102000007@hbut.edu.cn (Z.Y.); songxc@mail.hbut.edu.cn (X.S.); 2 School of Mechanical Science and Engineering, Huazhong University of Science and Technology, Wuhan 430074, China; yihuakang@hust.edu.cn

**Keywords:** magnetic permeability perturbation, non-destructive testing (NDT), magnetizing-based eddy current testing (MB-ECT), magnetic flux leakage (MFL), DC magnetization

## Abstract

DC magnetization is generally considered to suppress the usual local magnetic permeability variation and increase the penetration depth for magnetizing-based eddy current testing (MB-ECT) of ferromagnetic materials. In fact, such simple explanations lead to rough nondestructive evaluation and cause new neglected non-uniform magnetic characteristics. Hence, the “perturbation” of the internal magnetic field variation is analyzed using a magnetic dipole model and the mechanism of magnetic permeability perturbation in MB-ECT is revealed. The theoretical analysis and simulations show that a significant permeability perturbation always appears around a defect and presents opposite features with strong and weak magnetization. Furthermore, experimental results indicate that the hidden signal component arising from the local permeability perturbation is critical for both far-side surface and near-side surface defects in the MB-ECT method.

## 1. Introduction

Eddy currents have many applications in microwave heating and nondestructive evaluation for various metallic materials because of its easy realization, high sensitivity, and low cost [1,2,3,4,5]. Dr. Foster works to perfect eddy current testing (ECT) technology in theory and promotes the practical application of nondestructive evaluation in the industry worldwide [6]. With the development of the ECT method, considerable attention is focused on the theoretical model and analytical solutions in ECT [7,8,9], application research on new ECT probes [10,11,12,13], and signal inversion to reconstruct the defect [14,15].

It is widely accepted that the ECT method is primarily on the electromagnetic field disturbance caused by the conductivity change of a defect, but it is effective for defects in the surface layer of material due to the limited penetration depth of the eddy current. Therefore, for ferromagnetic materials, a direct current (DC) magnetization is always applied to the object and is called the MB-ECT method [16]. The DC magnetization is explained to reduce the “skin effect” by magnetic saturation of the wall and causes a lower permeability and a greater penetration depth of the eddy current [17]. Additionally, it also suppresses the usual local permeability variations in the material and eliminates an enormous source of noise [18]. A typical explanation is described in the standard ASTM E309-16: “the material under examination is effectively rendered nonmagnetic, this condition allows an eddy current system to measure and detect electrical resistivity and geometrical variations independent of concurrent variations in magnetic properties” [19]. In theoretical study, a simplified model with uniform magnetic properties is frequently used for a general analysis of electromagnetic detection. D.L. Atherton et al. apply the enhanced magnetization field to the regions of the excitation coil and the detection coil in the far-field eddy current testing. They recognized that the magnetic properties of the material changed, but they simply reduced the relative permeability of the material in the strong magnetization field from 70 to 5, according to the decreasing phase of the μ-H curve in the simulation model, without considering the real magnetic property change and its distribution [20]. Additionally, several studies present analytical solutions for an impedance change in eddy current testing with depth-varying magnetic permeability [21].

**Figure 1:**
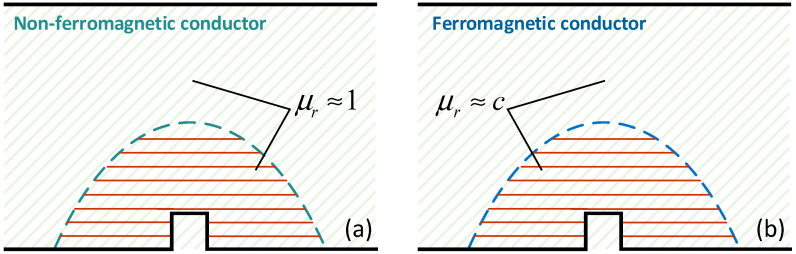
Traditional view of the magnetic permeability in the MB-ECT method. (**a**) non-ferromagnetic material, (**b**) ferromagnetic material.

In summary, researchers in both academia and engineering generally believe that magnetic permeability is evenly distributed in materials after magnetization; that is, the macroscopic discontinuities will not change the uniformity of the permeability distribution [22], as shown in Figure 1. Consequently, researchers seldom note the permeability variation in space caused by a defect in ferromagnetic materials. The “uniformity theory” about permeability is based on a uniform change of a magnetic field within a material; however, the internal magnetic field is strongly affected by a discontinuity under the DC magnetization and the permeability varies greatly with the level of the applied field. It is worth noting that the previous simple equivalent of permeability may result in insufficient MB-ECT measurements and applications.

In this paper, we further analyze the magnetic field perturbation caused by the defect in ferromagnetic materials based on a magnetic dipole model. Furthermore, we highlight that a permeability perturbation mechanism essentially exists in both the above-region and the side-region of the defect, which completely differs from the previous “uniformity theory” used in the present MB-ECT method. Finally, we primarily aim to reveal the non-negligible role of a permeability perturbation to establish a real MB-ECT theory system to obtain an accurate and comprehensive evaluation of MB-ECT technology.

## 2. Mechanism

In a magnetized state, the magnetic dipoles inside a material are connected end to end such that the positive and negative charges only appear on the cross section of the material, such as on the side of a defect. The property generated by the magnetic charges is often calculated by using a magnetic dipole model [23,24]. The opposite magnetic polarities with a line density of $\sigma_{s}$ are assumed to be uniformly distributed on the walls of a rectangle defect. The magnetic charges distributed over the length dy of the left sidewall of the defect are described as $dp=\sigma_{s}dy$. The magnetic field generated by the magnetic charges can be expressed by

(1) $$dH=\frac{dp}{4\pi r^{2}}\vec{r}$$

**Figure 2:**
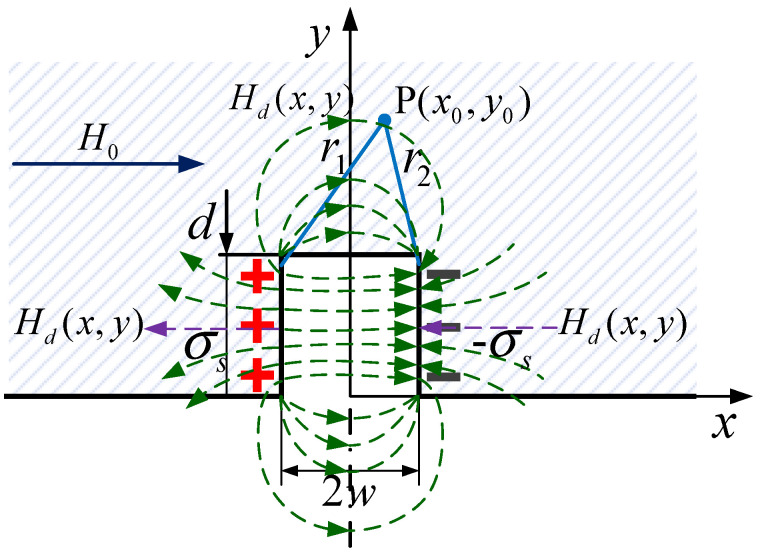
Magnetic field distribution based on the magnetic charges model.

As schematically illustrated in Figure 2, the magnetic field caused by the magnetic charges at point $P(x_{0},y_{0})$ can be calculated by

(2) $$\begin{array}{cc} H_{x}(x_{0},y_{0}) & =\int_{0}^{d}\frac{\sigma_{S}(x_{0}+w)}{2\pi [{\left(x_{0}+w\right)}^{2}+{\left(y_{0}+y\right)}^{2}]}dy+\int_{0}^{d}\frac{-\sigma_{S}(x_{0}-w)}{2\pi [{\left(x_{0}-w\right)}^{2}+{\left(y_{0}+y\right)}^{2}]}dy\\ \; & =\frac{\sigma_{S}}{2\pi }(\tan^{-1}\frac{d(x_{0}+w)}{{\left(x_{0}+w\right)}^{2}+y_{0}(y_{0}+d)}-\tan^{-1}\frac{d(x_{0}-w)}{{\left(x_{0}-w\right)}^{2}+y_{0}(y_{0}+d)}) \end{array}$$

It can be seen from Equation (2) that $H_{x}(x,y)$ is an even function and $H_{x}(x,y)$ decreases with an increase in |x|. The discontinuity orientation is perpendicular to the magnetization direction (the x direction), and the internal magnetic field of the material is primarily along x. In this case, the change in the x component is consistent with $H_{sum}(x,y)$. Therefore, we primarily analyze the x component of the magnetic field and use $H_{d}(x,y)$ to represent $H_{x}(x,y)$ in Figure 2.

**Figure 3:**
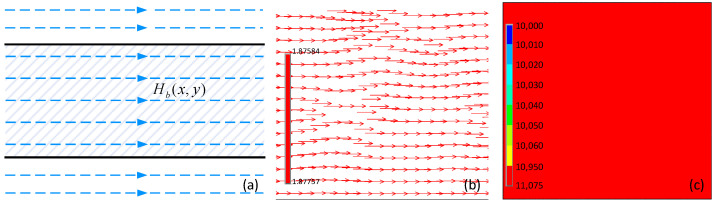
Dynamic magnetic action inside the material without any discontinuities, which is obtained by using the finite element method and magnetized by an encircling coil with a current density of 7 × 10^6^ A/m^2^. (**a**–**c**) show the magnetic field lines, the magnetic vector, and the magnetic contour, respectively.

The magnetic field in the material consists of not only the $H_{d}(x,y)$ caused by the magnetic charges presented in Figure 2 but also the original magnetic field without the slot $H_{b}(x,y)$. When there is no slot in the material, the magnetic field lines are in the same direction as the incident field lines outside the material. Thus, the magnetic flux travels directly through the interior of the material without any perturbation, as shown in Figure 3a,b, which were obtained by FEA. The magnetic field is distributed evenly throughout the material without any magnetic field perturbation, as indicated in Figure 3c.

**Figure 4:**
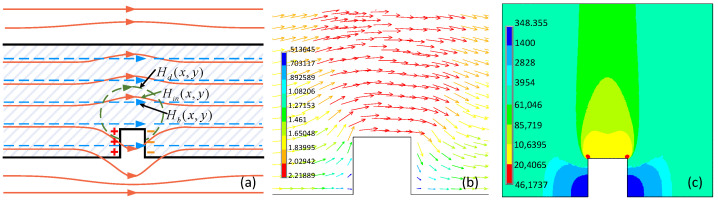
Dynamic magnetic action inside the material obtained by using the finite element method. The rectangular defect has the size of 1.2 mm (length) and1.5 mm (height). (**a**–**c**) shows the magnetic field lines, the magnetic vector, and the magnetic contour, respectively.

However, when there is a slot in the material, the magnetic flux travels through the interior of the material but is inhibited by the slot. The existing slot makes part of the magnetic flux twist abruptly to the region above it, while the remaining flux leaks out of the material into the vicinity of the crack, producing a magnetic flux leakage (MFL) [25]. The magnetic field tends to be channeled through the material, forming a magnetic flux perturbation phenomenon in the ferrous body, as shown in Figure 4a,b, which were obtained by FEA. The rectangular defect has the size of 1.2 mm (length) and1.5 mm (height). The magnetizer uses encircling coils with a current density of 7 × 10^6^ A/m^2^. Figure 4a–c shows the magnetic field lines, the magnetic vector, and the magnetic contour, respectively. As a result, the internal magnetic field is distributed unevenly throughout the material with the magnetic field perturbation, as indicated in Figure 4c.

**Figure 5:**
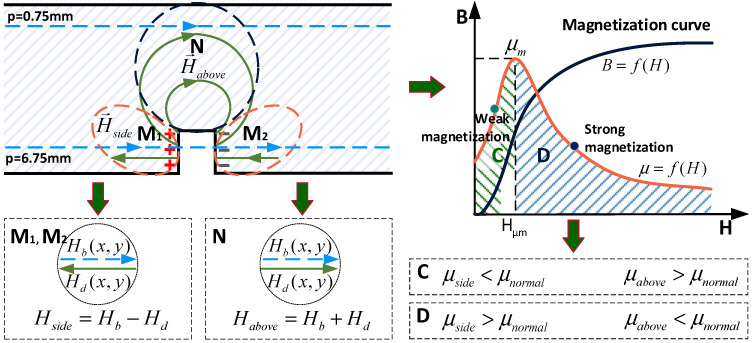
Mechanism of permeability perturbation hidden in the present magnetizing-based eddy current testing.

According to the magnetic contour in Figure 4c, the magnetic field of the regions around the defect provide notably different features; the magnetic field above the defect is stronger, while the magnetic field at the sides of the defect is weaker. The magnetic field perturbation is analyzed in detail and presented in Figure 5, where three different regions are considered, namely, region M_1_, region M_2_, and region N. $H_{b}(x,y)$ and $H_{d}(x,y)$ are the original magnetic field without a defect and the magnetic field caused by the magnetic charge, respectively. $H_{side}$ is the magnetic fields of regions M_1_ and M_2_. $H_{above}$ is the magnetic fields of regions N. $\mu_{above}$, $\mu_{side}$, and $\mu_{normal}$ are the permeability of regions M_1_ and M_2_, the permeability of region N, and the permeability of the region far from the crack, respectively. The magnetic charges produce a negative magnetic field in regions M_1_ and M_2_ where the direction of $H_{b}(x,y)$ is in the opposite direction of $H_{d}(x,y)$ in the material. On the contrary, the magnetic charges produce a positive magnetic field in region N, where $H_{b}(x,y)$ and $H_{d}(x,y)$ are in the same directions. Furthermore, regions M_1_ and M_2_ result in an enhanced $H_{side}$, while region N is characterized by a weakened $H_{above}$, as presented in Figure 5.

(3) $$\{\begin{array}{cc} H_{side}=H_{b}-H_{d} & region{\;M}_{1}\;and{\;M}_{2}\\ H_{above}=H_{b}+H_{d} & region\;N \end{array}$$

From the perspective of the physical properties, the nonlinear permeability $\mu_{f}$ (μ=f(H)) of a ferromagnetic substance reaches a maximum and later declines due to saturation, as shown in Figure 5 [26]. The complete μ-H curve of the ferromagnetic material is divided into ascending and descending stages according to the peak $\mu_{m}$.

In the ascending stage,

(4) $$\mu_{side}>\mu_{normal}$$

(5) $$\mu_{above}<\mu_{normal}$$

In the descending stage,

(6) $$\mu_{side}<\mu_{normal}$$

(7) $$\mu_{above}>\mu_{normal}$$

Therefore, the magnetic permeability perturbation caused by the far-side surface defect not only occurs in the space above the defect, but also occurs in the side space of the defect, and a non-uniform distribution of magnetic permeability is formed inside the material. The phenomenon of this non-uniform permeability distribution is called the “permeability perturbation”. It is worth noting that the permeability of a material varies with the magnetization state of the material. This does not mean that the properties of the material have actually changed, but moved to different points on the μ-H curve.

**Figure 6:**
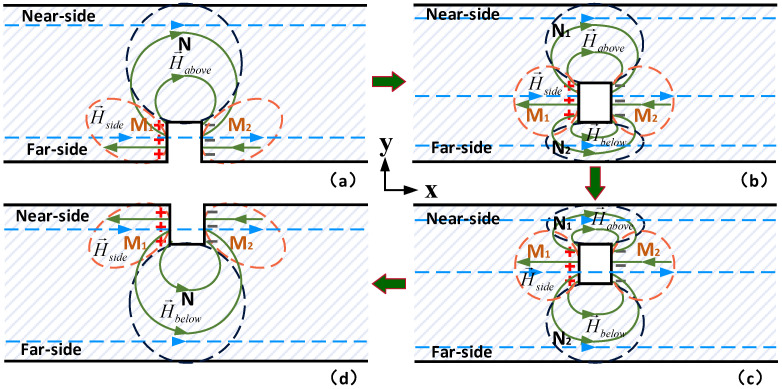
Schematic diagram of magnetic field perturbation of defects of different buried depths. (**a**) far-side surface defect; (**b**,**c**) internal non-surface opening defects; (**d**) near-side surface defect.

The above analysis is based on the far-side surface defects, but the magnetic permeability perturbations appear not only in far-side surface defects but also in the internal defects (not surface breaking defect) and near-side surface defects, so the location of the defects is further considered, as shown in Figure 6. The defects are located inside the material and do not touch the upper and lower surfaces, as shown in Figure 6b,c. The magnetic charge generating magnetic field forms the magnetic field perturbation in the regions above the defect and the region below the defect, which are recorded as the N_1_ and N_2_. In the left and right regions of the defect, the magnetic field perturbation is symmetrically distributed about the centerline of the defect. However, in the upper and lower regions of the defect, due to the limitation of the diffusion range, the magnetic field perturbation changes with the buried depth of the defect. As the buried depth of the defect is gradually reduced, it eventually evolves into the near-side surface defect, as shown in Figure 6d. At this time, the magnetic field perturbation regions M_1_ and M_2_ move to the vicinity of the near-side surface, and the magnetic field perturbation region N moves to the region directly below the defect. In the process of (a–d) shown in Figure 6, the magnetic permeability perturbation regions M_1_ and M_2_ move upward in the *y* direction while the N region is divided into N_1_ and N_2_, eventually moving from the top of the defect to the bottom of the defect in the *y* direction.

## 3. Simulation and Experiment

### 3.1. Verification via FEM

**Figure 7:**
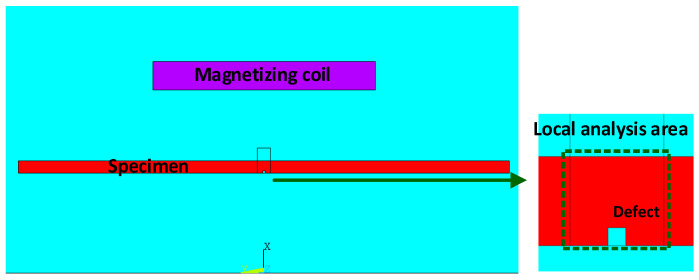
The 2D axisymmetric model.

**Figure 8:**
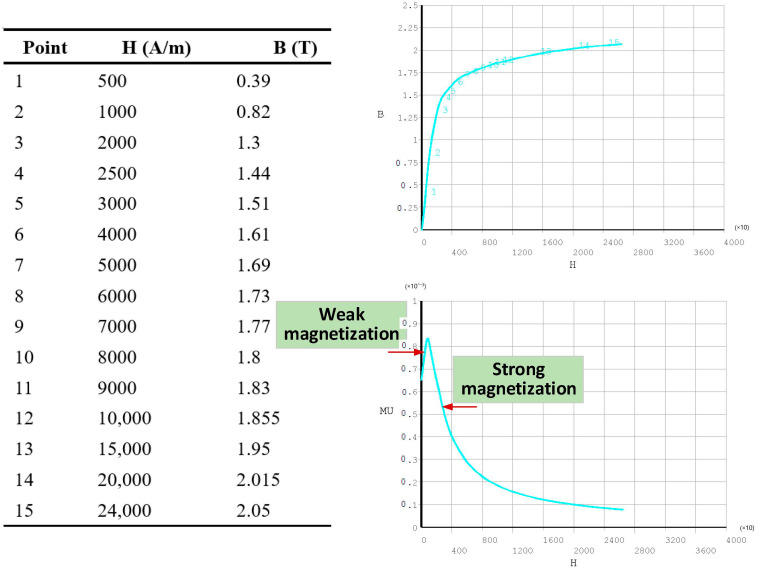
B-H curve of the material used in ANSYS.

The ANSYS software (Mechanical APDL) was used for the FEM analysis. A 2D axisymmetric model is built to analyze the magnetic permeability perturbation around the defect, as shown in Figure 7. The local refinement is used to make the solution results more accurate. The wall thickness used for the simulation is 7.5 mm. The B-H curve of the No.45 steel in GB (Chinese National Standards) used in ANSYS is shown in Figure 8.

**Figure 9:**
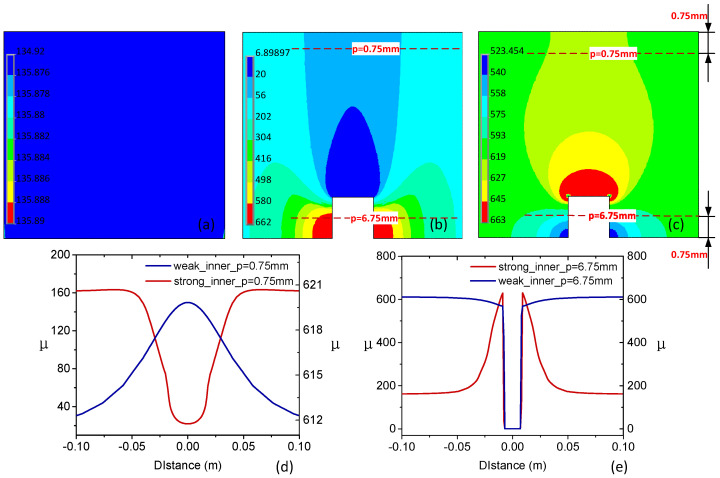
Relative permeability distribution obtained by simulations. (**a**–**c**) show the magnetic permeability distribution under no-defect, strong magnetization, and weak magnetization, respectively. (**d**,**e**) represent the permeability change curves of the path *p* = 0.75 mm and the path *p* = 6.75 mm.

The permeability perturbation around the defect is obvious compared with plots of materials without any defects in Figure 9a. This magnetic permeability perturbation is similar to a “bubble” shape, and the magnetic permeability is not a simple one-way perturbation, but rather a bubble-like distribution diffusion exists. It is difficult to describe this difference by means of cloud image display. Therefore, by calculating and extracting the permeability information on the path to be analyzed, the characteristics of magnetic permeability perturbation can be described more accurately.

The weak magnetization corresponds to the rising phase (C) of the magnetization curve in Figure 5 and the strong magnetization corresponds to the decreasing phase (D) of the magnetization curve in Figure 5. The corresponding current densities under strong magnetization and weak magnetization are 1.2 × 10^6^ A/m^2^ and 7 × 10^6^ A/m^2^, respectively. It can be seen that the defect produces a positive magnetic field and forms the “convex” feature of permeability under weak magnetization and the “concave” feature under strong magnetization as expected. In contrast, the permeability change curves of the path *p* = 6.75 mm (Figure 5) show a “concave” feature in both cases, as shown in Figure 9e, as a result of the electromagnetic properties of the crack. The curve under strong magnetization shows a “double-peak” feature, resulting from the negative magnetic field and an increase in the permeability of region A. The noted permeability perturbation arising from the defect is quite different from the well-known one, i.e., the DC magnetization suppresses the local permeability variations in the material and promotes the uniform distribution of the magnetic permeability inside the material.

**Figure 10:**
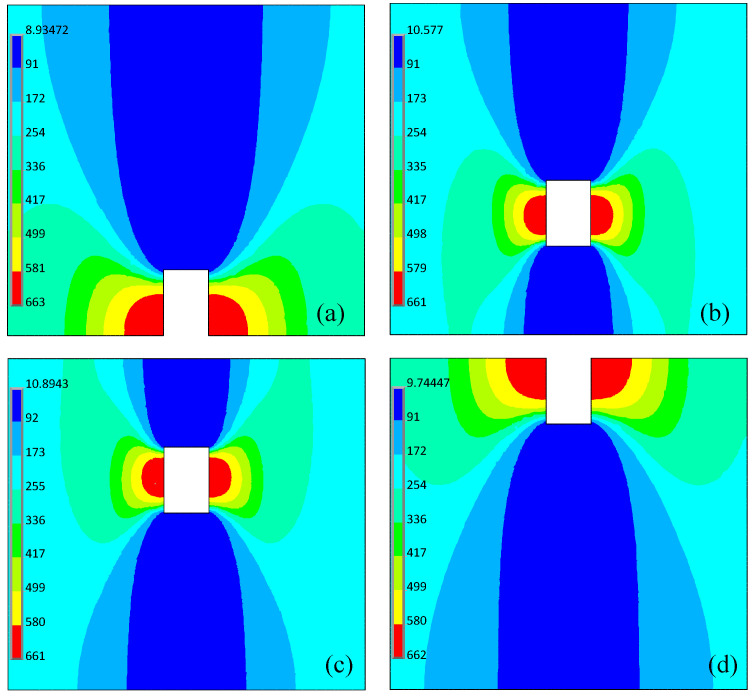
Permeability cloud map of different buried depth defects. (**a**) far-side surface defect; (**b**) and (**c**) internal non-surface opening defects; (**d**) near-side surface defect.

The finite element analysis of the defects with different buried depths is performed to obtain the magnetic permeability distribution cloud around the defect, as shown in Figure 10. Consistent with the previous analysis, magnetic permeability perturbation occurs around the defects of different buried depths. For the far-side surface defect, the magnetic permeability perturbation mainly occurs on the side regions and above region of the defect and spreads to the surface layer of the material. It is worth noting that in Figure 10b,c, magnetic permeability perturbations are formed in the regions around the internal defects, especially above and below the defects, which spread to the near-side surface and the far-side surface of the material. For the surface defect, the magnetic permeability perturbation region above the defect gradually disappears, but the magnetic permeability perturbation regions on both sides of the defect move to the surface layer to form a discontinuity region with the defect.

### 3.2. Verification via Experiments

**Figure 11:**
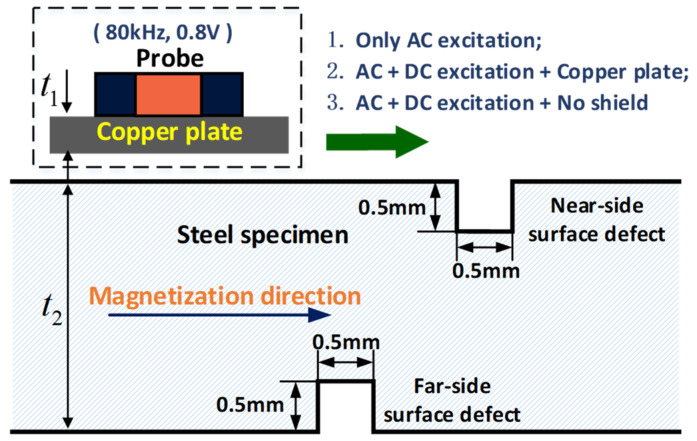
Experimental diagram of MB-ECT. $t_{1}$ and $t_{2}$ represent the thickness of the copper plate and the specimen, respectively.

In this section, a set of experiments are performed on the steel plates to verify the effect of the magnetic permeability perturbation by steps 1–3, as shown in Figure 11. The specimens were 45# steel plates with different thicknesses $t_{2}$. The widths of the steel plates are both 100 mm and the thicknesses are 7.5 mm and 11.5 mm, respectively. The rectangular longitudinal section cracks were produced on the positive side and opposite side of the specimens, and all the cracks were machined by electric discharge machining. The DC magnetic field is provided by a magnetizer based on only one encircling coil (2000 turns) with a current of I = 16 A. The inner diameter and the outer diameter of the encircling coil are 140 mm and 174 mm.

The probe consists of a single excitation coil and two differentially connected receiving coils. The inside diameter and outside diameter of the coils are 3 mm and 4 mm, respectively. The excitation coil is wound with 50 turns of 0.13 mm copper wire, the dc resistance and inductance of which are 2.4 Ω and 11.819 μH, respectively. The receiving coil is wound with 150 turns of 0.05 mm copper wire, the dc resistance and inductance of which are 15.7 Ω and 89 μH, respectively. The AC excitation voltage is 0.8 V and the operating frequency is 80 kHz. A copper plate with a thickness of $t_{1}$ is used to shield the eddy current field in some steps.

**Figure 12:**
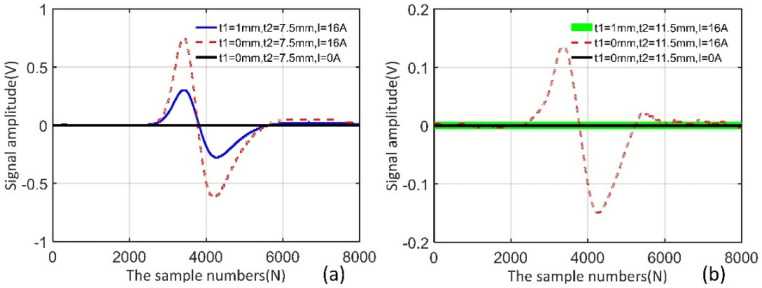
Experimental results. (**a**) far-side surface defect of a 7.5 mm thick specimen, (**b**) far-side surface defect of an 11.5 mm thick specimen.

As shown in Figure 12a,b, the far-side surface defect cannot be detected by only the AC excitation, owing to the skin effect of the eddy current; meanwhile, the signal amplitude is significantly reduced but not disappeared as in the case of the shield ($t_{1}$ = 1 mm), where there exists both the AC and DC excitation. In the MB-ECT method, several magnetic lines of force pass through the specimen and exit out of the positive side, which is a mutated disturbed field compared to the moving induction coil. The copper shield does not affect the MFL signal, but it affects the AC excitation as a result of the skin effect under the AC magnetic field.

For a thicker specimen ($t_{2}$ = 11.5 mm), the magnetic flux leakage is too small to be detected due to the magnetic shielding of the wall despite the fact that the DC magnetization is applied to the specimen. In addition, the copper shield affects the AC excitation owing to its skin effect and there is no response from the far-side surface defect. In addition, when the thickness of the material increases, the permeability perturbation in the surface layer of the material becomes weaker, which results in a smaller testing signal.

The skin depth is about 0.347 mm based on μ = 10 and σ = 3.3 e7 S/m (conductivity). The skin depth is sufficiently small enough that the far side is more than 3δ (the effective inspection depth). For the far-side surface defect, the permeability perturbation above the defect causes the eddy current perturbation. However, the near-side surface defect and the permeability perturbation on both sides of the defect all have an effect on the eddy currents in the surface layer. In fact, the two factors work together under DC magnetization and cannot be separated, which makes it impossible to determine the signal source from a single set of signals. Therefore, when we conduct experiments on the near-side surface defect, the relationship between the signal amplitude and the magnetizing current is mainly considered.

**Figure 13:**
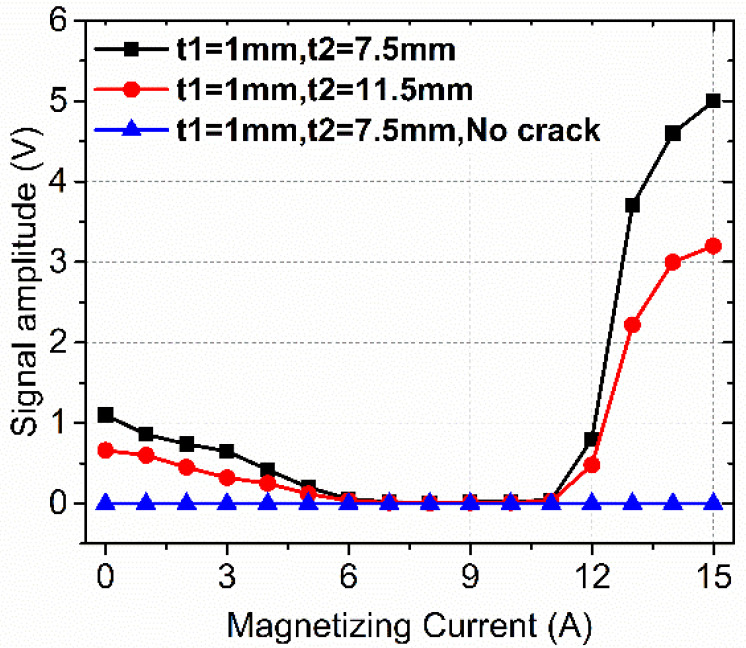
The relationship between signal amplitude and magnetizing current.

Figure 13 shows the detection results of near-side surface defect. As shown in Figure 13, it can be clearly noticed that there is a gradual decline in the signal amplitude and then it increases, presenting the “concave” feature. Actually, the MFL increases with the magnetization current [27]. However, the equivalent current source of the defect would not be affected by the magnetization, and therefore the permeability perturbation in the side-region accounts for the “concave” feature and it is not always beneficial for detection. To conclude, not only does the proposed permeability perturbation have an effect on the far-side surface defect, but it also acts as the signal source for the near-side surface defect.

## 4. Discussion

**Figure 14:**
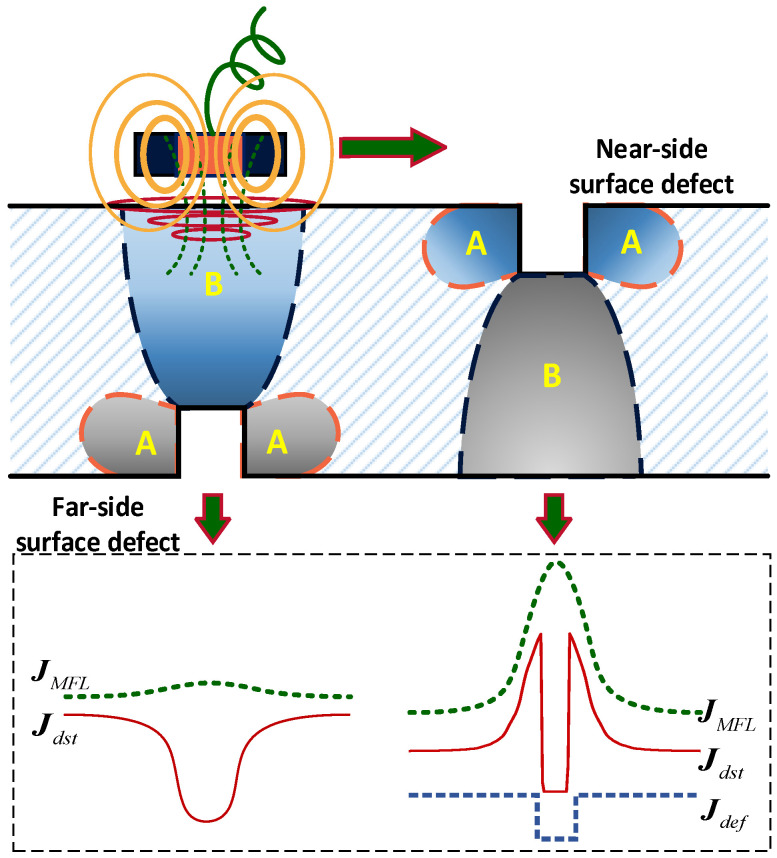
Signal source of the MB-ECT method for a far-side surface defect and a near-side surface defect.

Based on the previous analysis, the permeability perturbation always appears with the existence of defects, including a near-side surface defect and a far-side surface defect, as shown in Figure 14. According to the equivalent source method, the defect is equivalent to a current source and the disturbed field caused by the crack is equivalent to a field due to the equivalent current source. In the MB-ECT method, the total disturbed field mainly consists of the disturbed field caused by the crack, the disturbed field caused by the permeability distortion, and the magnetic leakage field, which is equivalent to the current source $J_{mag}$, as described in Equations (8) and (9) [28]. Actually, the skin effect always limits the eddy current to reach the far-side surface defect (buried depth > three times penetration depth) even if the permeability decreases to one, and then Equation (5) can be expressed as Equation (10).

(8) $$\nabla \times H_{def}=\sigma E_{def}+J_{mag}$$

(9) $$J_{mag}=J_{def}+J_{dst}+J_{MFL}$$

(10) $$J_{mag}=J_{dst}+J_{MFL}$$

where $J_{mag}$ is the total equivalent eddy current density, $J_{dst}$, $J_{def}$, and $J_{MFL}$ are, the equivalent current density due to the permeability perturbation, the crack itself, and the magnetic flux leakage, respectively.

The source of $J_{dst}$ for the near-side surface defect is different from the source of the far-side surface defect. The permeability perturbation of region A plays a leading role for the near-side surface defect, while the permeability perturbation of region B is the main signal source for the far-side surface defect owing to the skin effect of the eddy current.

## 5. Conclusions

In summary, the DC magnetization for ferromagnetic materials in MB-ECT method is not as simple as traditional descriptions such as “the magnetization suppresses the magnetic characteristic anomaly and increases the penetration depth”. From the perspective of the magnetic dipole, the additional magnetic field produced by magnetic charges attached to the defect result in local magnetic field perturbations. In this case, the permeability perturbation in the vicinity of a defect is primarily related to the magnetic field perturbation in the material as a result of the nonlinear µ-H curve. Hence, it is extremely unwise to make such a hypothesis in the standard that the material under DC examination can be equivalent to homogeneous media in the MB-ECT method. The permeability in the above-region and side-region of the defect always change in opposite directions due to the negative magnetic field and positive magnetic field produced by the magnetic charges. The permeability perturbation always appears along with the existence of defects, including the near-side surface defects such as cracks, pits, and corrosions, and the far-side surface defects, such as corrosions and eccentric wears. Different perturbation features present under different magnetization fields and act as an important disturbed source to the magnetic sensors. Furthermore, for a deeper defect, the permeability perturbation around the defect spreads to the surface layer of the material and is detected by the sensors, forming a new detection method named the permeability perturbation testing (MPPT). The clarity and the discovery of the mechanism of permeability perturbation and its effects are of benefit for enriching the theoretical system and enhancing the evaluation accuracy of the MB-ECT method.
